# An Integrative Transcriptomic Analysis for Identifying Novel Target Genes Corresponding to Severity Spectrum in Spinal Muscular Atrophy

**DOI:** 10.1371/journal.pone.0157426

**Published:** 2016-06-22

**Authors:** Chung-Wei Yang, Chien-Lin Chen, Wei-Chun Chou, Ho-Chen Lin, Yuh-Jyh Jong, Li-Kai Tsai, Chun-Yu Chuang

**Affiliations:** 1 Department of Biomedical Engineering and Environmental Sciences, National Tsing Hua University, Hsinchu, Taiwan; 2 Stroke Center and Department of Neurology, National Taiwan University Hospital, Taipei, Taiwan; 3 Graduate Institute of Clinical Medicine, Kaohsiung Medical University, Kaohsiung, Taiwan; 4 Departments of Pediatrics and Clinical Laboratory, Kaohsiung Medical University Hospital, Kaohsiung Medical University, Kaohsiung, Taiwan; 5 Department of Biological Science and Technology, National Chiao Tung University, Hsinchu, Taiwan; University of Edinburgh, UNITED KINGDOM

## Abstract

Spinal muscular atrophy (SMA) is an inherited neuromuscular disease resulting from a recessive mutation in the *SMN1* gene. This disease affects multiple organ systems with varying degrees of severity. Exploration of the molecular pathological changes occurring in different cell types in SMA is crucial for developing new therapies. This study collected 39 human microarray datasets from ArrayExpress and GEO databases to build an integrative transcriptomic analysis for recognizing novel SMA targets. The transcriptomic analysis was conducted through combining weighted correlation network analysis (WGCNA) for gene module detection, gene set enrichment analysis (GSEA) for functional categorization and filtration, and Cytoscape (visual interaction gene network analysis) for target gene identification. Seven novel target genes (*Bmp4*, *Serpine1*, *Gata6*, *Ptgs2*, *Bcl2*, *IL6* and *Cntn1*) of SMA were revealed, and are all known in the regulation of *TNFα* for controlling neural, cardiac and bone development. Sequentially, the differentially expressed patterns of these 7 target genes in mouse tissues (e.g., spinal cord, heart, muscles and bone) were validated in SMA mice of different severities (pre-symptomatic, mildly symptomatic, and severely symptomatic). In severely symptomatic SMA mice, *TNFα* was up-regulated with attenuation of *Bmp4* and increase of *Serpine1* and *Gata6* (a pathway in neural and cardiac development), but not in pre-symptomatic and mildly symptomatic SMA mice. The severely symptomatic SMA mice also had the elevated levels of *Ptgs2* and *Bcl2* (a pathway in skeletal development) as well as *IL6* and *Cntn1* (a pathway in nervous system development). Thus, the 7 genes identified in this study might serve as potential target genes for future investigations of disease pathogenesis and SMA therapy.

## Introduction

Spinal muscular atrophy (SMA) is an autosomal recessive neuromuscular disease involving low expression of survival motor neuron (SMN) protein due to homozygous mutation of the *SMN1* gene [1]. SMA is one of the most common inherited causes of infant mortality, with an incidence of 1 in 10000 live births and a carrier frequency of 1 in 31 [2]. SMA is categorized as type I—type IV according to the age of onset and disease severity. The main pathological features of SMA include neuron loss in the anterior horn of the spinal cord, atrophy of skeletal muscle [3], and functional and structural disruption of synaptic connectivity at neuromuscular junctions [4, 5]. The molecular pathogenesis of SMN deficiency is still unclear, and there is no disease-modifying therapy currently available for SMA patients.

Two *SMN* genes have been identified in humans: the telomeric *SMN1* encoding full-length SMN protein, and its centromeric homolog, *SMN2*, generating primarily rapidly-degraded SMN protein lacking exon 7 [6]. Since 10% of the *SMN2* gene product is full-length SMN protein, *SMN2* expression may compensate for insufficient production of mutated *SMN1*. Indeed, the *SMN2* copy number correlates negatively with disease severity [7, 8]. Thus, *SMN2* is considered as an important SMA disease modifier to be a potential target for SMA treatment. However, the correlation between *SMN2* copy number and clinical phenotype does not apply to all SMA patients, and medications endeavoring to increase SMN production only slightly attenuate the disease symptoms [9, 10]. Other genetic modifiers, plastin 3 *(Pls3)*, zinc finger protein 1 (*Zpr1*) and ubiquitin-like modifier activating enzyme 1 (*UBA1*), have been identified based on their biological functions and genotype-phenotype prevalence in SMA [11–13]. Although these modifiers provide some clues for understanding genetic regulation in SMA, treatments using these modifiers as therapeutic targets have been either ineffective or limited [14, 15]. In addition, SMA is a multi-system malfunctioned disease with wide spectrum of disease severity [16, 17]. Treatments dedicated to motor neuron function have shown some therapeutic effects; however, none has demonstrated substantial curative response in SMA patients due to the disruption of pathologies outside the CNS such as metabolic disorders and dysfunctional autonomic innervation of the heart [10, 18, 19]. Successful development of new SMA therapies requires the identification and characterization of pathological changes that occur across different cell types and tissues affected by SMA [16]. An appropriate bioinformatics analysis such as using microarray datasets from publicly available databases is one of the most favorable methods for retrieving critical information across different cells or tissues with fewer expenses and more diversity.

Several SMA microarray studies have used traditional gene selection method based on significance testing to identify highly differentially expressed genes (DEGs) in revealing pathogenic mechanisms or targets for potential treatments with inconsistent results [13, 20]. Recently it has been found that the method using highly connected hub gene selection usually generates more meaningful gene lists than standard statistical analysis in several biological issues [21]. In the present study, we conducted an integrative transcriptomic analysis across different cell types to identify potential SMA targets that can be generally applied to the alteration in multi-systems using weighted correlation network analysis (WGCNA) for calculating suitable module genes highly correlated to the clinical severity of SMA, gene set enrichment analysis (GSEA) for filtrating module genes with unknown functions, and Cytoscape for reconstructing crucial molecular networks and novel candidate genes in the regulation of SMA pathogenesis. With the determination of gene expression patterns in four tissues of SMA model mice, these target genes were validated to be critically relevant to disease severity of SMA, which can be used as potential SMA modifiers for future therapy.

## Materials and Methods

### Data collection and preprocessing

This data re-analysis procedure was approved by the institutional review board of National Tsing Hua University. All of the datasets used in this study were processed by the contributors to anonymize any records of patients before submitting to ArrayExpress and GEO databases. The criteria of dataset selection in this study were (1) human samples with certified SMA types and age-matched controls, and (2) no any treatment procedure was applied. All of the selected microarray datasets were assumed to be equivalent and the hybridization and processing conditions were confident for further analysis. There were 39 microarray datasets recruited from three individual high-throughput experiments of functional human SMA genomics in four distinct human cells (iPS: 12; iPS differentiated motoneurons: 6; fibroblast cells: 3; muscle cells: 18) from ArrayExpress (E-GEOD-27207: HG-U133 plus 2 and E-GEOD-13828: Human Exon 1.0 ST) and Gene Expression Omnibus (GEO; GSE8359) databases of NCBI (Table 1). The datasets E-GEOD-27207 (type I SMA vs. control) and E-GEOD-13828 (type I SMA vs. control) were individually normalized using robust multi-array average (RMA) method that is a standard quantile normalization for making the distribution of probe intensities comparable between different arrays [22]; otherwise, the dataset GSE8359 (type I SMA vs. type III SMA) has been normalized by the designers before uploaded to GEO. To attenuate the batch effect from various handling procedures, the normalized E-GEOD-27207 and E-GEOD-13828 datasets were merged together according to their similarity in data structures, and were further cross-platform normalized using median rank scores (MRS) method of CONOR package in Bioconductor software. The duplicate probe sets of genes in each microarray dataset were averaged into one value to avoid aberration. The probe sets with no “gene name” or “entrez id” was excluded in the further analysis.

**Table 1:** Characteristics of public microarray datasets used in this study.

Study [Ref]	Species (Platform)	Tissues	Number of samples
E-GEOD-27207 [23]	Human (Affymetrix)	Induced pluripotent stem cells (iPS)	Type I SMA = 2, control = 4
		iPS differentiated motoneurons	Type I SMA = 3, control = 3
		Fibroblast cells	Type I SMA = 2, control = 1
E-GEOD-13828 [N/A]	Human (Affymetrix)	Induced pluripotent stem cells	Type I SMA = 1, control = 5
GSE8359 [20]	Human (Italian lab)	Muscle cells	Type I SMA = 8, type III SMA = 10

E-GEOD: ArrayExpress dataset; GSE: GEO dataset

According to SMA types and data structure, two groups, type I SMA *vs*. control (SI/C) and type I SMA *vs*. type III SMA (SI/SIII), were categorized to unveil the cause of severity in SMA. SI/C (E-GEOD-27207 and E-GEOD-13828) compared the differences between type I SMA patients and controls; SI/SIII (GSE8359) focused on the differences between type I and type III SMA patients. These two groups were analyzed independently to find out hub genes representing the cause of severity between different stages, and the hub genes in both groups were intersected to indicate candidate SMA targets. The expression of ± 1.2-fold change between SMA patients and controls were used to select DEGs in SI/C. As to SI/SIII, since GSE8359 is a muscle specific microarray with fewer probe sets, all of the genes were retained to average the same probe sets and processed to the following analysis in R-studio statistical software.

### Weighted gene co-expression network analysis (WGCNA) of module identification in SI/C and SI/SIII groups

According to the protocol of WGCNA [24], the adjacency matrices were calculated by raising the absolute values of the weighted correlation matrices to a power of six, and the topological overlap (TO) for the node similarity was measured for exploring suitable modules. For the initial module identification, genes were hierarchically clustered using 1−TO as the distance measurement and modules were determined by using a dynamic tree-cutting algorithm. Each gene’s module membership (MM) for a given module was then estimated as the average Pearson correlation between the gene and another five genes in a module with the highest within-module connectivity (kin), which presented a good approximation of the module eigengene (ME). P values were obtained by (1) averaging the T-score from gene-eigengene Pearson correlation across datasets, (2) scaling to the square root of the number of datasets per gene, and (3) calculating a P value from the T-distribution of the resulting scaled T-score. For the final module characterizations, all genes with MM values of R > 0.2 and with P < 10^−13^ were assigned to a module.

### Gene set enrichment analysis (GSEA) of gene ontology in SI/C and SI/SIII groups

Total genes of SI/C (213 genes) and SI/SIII (157 genes) from the selected modules were independently processed in GSEA to be clustered into gene ontology (GO). In general, FDR q-value below 0.05 overall within GO databases were set as a limitation for ontology selection. Many databases can be used according to different users and study goals; we chose BIOCARTA, KEGG, REACTOME, BP (GO biological process) in this study for ontology distribution. To avoid any artificial judgment in selecting certain types of genes, the genes not involved in one of the ontologies were omitted.

### Network analysis of selected module genes

After omitted genes with no matched known ontology, there were 137 genes left in SI/C and 107 genes in SI/SIII, respectively. These processed genes of GSEA in SI/C and in SI/SIII were separately imported into Cytoscape 3.0.2 to reconstruct gene networks using ClueGO+CluePedia plug-in software. REACTOME and BP were used as reference databases, and the parameters were adjusted as the follows. GO term restriction was set at 4% of genes with P-value less than 0.05, which means that each gene cluster contains more than 4% of genes within a function with significant gene-gene correlation. GO term connection restriction (kappa score) was set at 0.4 as default setting.

### SMA mice

SMA model mice were generated by deletion of exon 7 of the *Smn* gene and knock-in of the human *SMN2* (*Smn*^*-/-*^*SMN2*^*+/-*^) [25]. We have since generated a variant that presents with more severe disease through back-crosses to obtain a more homogenous genetic background. This more severe mouse model of SMA (type I SMA mice) harbors two copies of *SMN2* transgene (*Smn*^*-/-*^*SMN2*^*+/-*^) with an average lifespan of 8.7 days [26]. We also generated a milder type of mouse model of SMA (type III SMA mice), which had 2 alleles of the *SMN2* transgenes (*Smn*^*-/-*^*SMN2*^*+/+*^). These type III SMA mice developed motor neuron degeneration since 6 months of age and could survive for more than 12 months [27]. Mouse genotypes were confirmed by PCR analysis as previously described [26]. All of the animal experiments were performed under protocols approved by the Utilization Committee and the National Taiwan University Institutional Laboratory Animal Care and Use Committee (IACUC # 20110468). Mice were under the care of the laboratory animal center of the National Taiwan University College of Medicine, and were supplied with sterile water and rodent pellets *ad libitum*. The method of sacrifice was conducted through the intraperitoneal injection of 150 mg/kg of pentobarbital.

After sacrificing mice, tissues were rapidly removed for RNA extraction (spinal cord, heart, muscle and bone) and neuronal density analysis (spinal cord). Spinal cords for neuronal density calculation were fixed in 4% paraformaldehyde at room temperature for 3 hours, followed by 15% sucrose for 1 day and 30% sucrose at 4°C overnight, and then were rapidly frozen in liquid nitrogen-cooled isopentane. Frozen serial sections of spinal cord were cut transversely at a thickness of 10 μm throughout the level of lumbar spinal cord, and stained with H&E as previously described [27]. The samples were observed, and images were captured digitally with a light microscope (Leica DM RA, Leica Microsystems, Germany). Neurons were identified according to their pyramidal shape, large nucleus and prominent nucleolus. We calculated the number of neurons (only neurons showing nuclei with their cell size of > 400 μm^2^) in the anterior horn.

**Fig 1:**
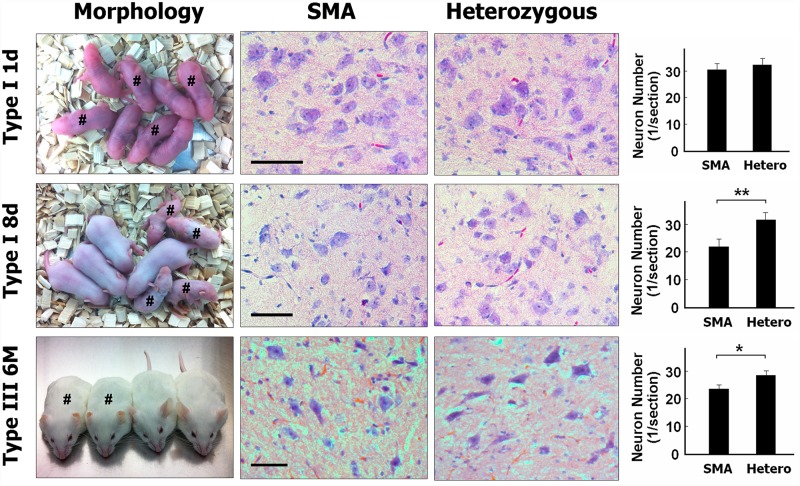
The appearance and density of lumbar spinal cord neurons in type I and III SMA mice. Type I SMA mice on postnatal day 1 had a body size and neuronal density similar to that of age-matched heterozygous control littermates. On postnatal day 8, type I SMA mice had a smaller body size and lower neuron density than age-matched heterozygous control littermates. At the age of 6 months, type III SMA mice displayed shorter tails, irregular ear borders, and a mild decrease in neuronal density compared to age-matched heterozygous control mice. Bar = 100 μm; #, SMA mice; n = 4 for each group; *P < 0.05; **P < 0.01.

Considering the disease spectrum of SMA, this study used SMA I and SMA III mouse models representing the situation in human patients to evaluate specific gene expression at various disease severity and stages (Fig 1): (1) Type I SMA mice on postnatal day 1 (pre-symptomatic stage), having similar body size and neuronal density in spinal cord as compared to those in age-matched heterozygous control littermates (*Smn*^*+/-*^*SMN2*^*+/-*^), (2) Type I SMA mice on postnatal day 8 (severe symptomatic stage), having smaller body size and lower spinal neuronal density than age-matched heterozygous controls littermates, and (3) Type III SMA mice at the age of 6 months (mild symptomatic stage), having shorter tail, irregular ear border, and mild decrease in spinal neuronal density as compared to those in age-matched heterozygous controls.

### Quantitative real-time PCR for determining mRNA expression

The gene expression of candidate targets identified from analysis was determined in different disease severity and stages of SMA mouse models. The RNA from the mouse whole spinal cord, heart, muscle, and bone was extracted using the Trizol reagent (Invitrogen, Carlsbad, CA). Total RNA was reverse-transcribed to cDNA using the high capacity cDNA reverse transcription kits (Applied Biosystems, Foster City, CA) following by quantification of gene expression using quantitative real-time PCR. PCR primers (TNFα: F-5’TCT GTG AAG GGA ATG GGT GTT-3’, R-5’TCT TGT GTT TCT GAG TAG TTG TTG A-3’; Bmp4: F-5’CGT GGG CTG GAA TGA TTG GAT-3’, R-5’ATG GTT GGT TGA GTT GAG GTG AT-3’; Serpine1: F-5’GGC ATA CGG CAT CCC ATT TG-3’, R-5’CAT CAC GAG GCT ACC CAG AG-3’; Gata6: F-5’AAA ACG CCA ACC CCG AGA AC-3’, R-5’GCC AGA GCA CAC CAA GAA TCC-3’; Ptgs2: F-5’TGC CTG GTC TGA TGA TGT ATG C-3’, R-5’AGT ATG AGT CTG CTG GTT TGG A-3’; Bcl2: F-5’CAG GTC TGT TGG GAG TGG TAT C-3’, R-5’TCA GGC TGG AAG GAG AAG ATG C-3’; IL6: F-5’CTT CCA TCC AGT TGC CTT CTT G-3’, R-5’CAG GTC TGT TGG GAG TGG TAT C-3’; Cntn1: F-5’CGG AAG TGA AGG TGA AGG AAG G-3’, R-5’TCT GGG AAG TGG TAG GGA GGA T-3’) were designed using Primer 6 software according to the mRNA sequence in GenBank. A Real-time PCR was performed with 2X power SYBR green PCR master mix (Applied Biosystems) in a sequence detector (Model 7300, Applied Biosystems). The relative level of mRNA expression to endogenous housekeeping gene GAPDH was normalized using comparative method by SDS 1.4 software. All of the experiments were at least triplicated in this study. Each condition consisted of 3–5 mice. Two-tailed P values of less than 0.05 were considered statistically significant.

## Results

### Ontological network reconstruction of SI/C and SI/SIII

**Fig 2:**
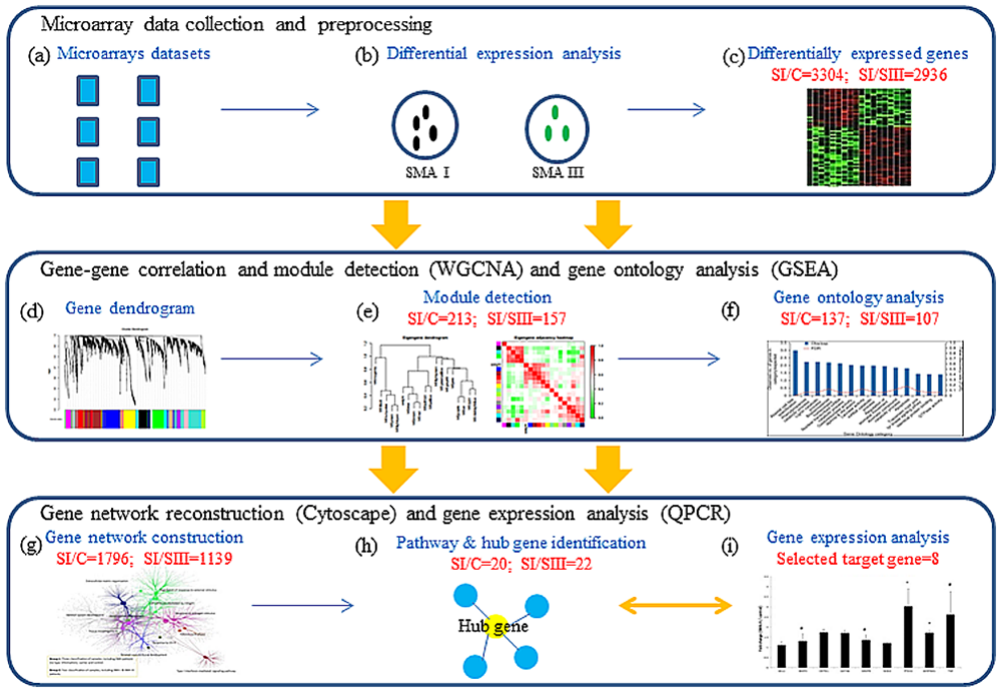
Schematic framework of target gene identification in SMA pathogenesis. **(a-c)** 3304 and 2936 DEGs with fold change of ±1.2 and significance were identified respectively in collected microarray datasets of type I SMA vs. controls (SI/C; n = 21) and type I vs. type III SMA (SI/SIII; n = 18) for potential target genes in SMA. **(d-e)** WGCNA is a systems biology method for describing the correlation patterns among genes across microarray samples [28]. These DEGs were processed into WGCNA to investigate 213 and 157 module genes highly correlated with the external information of classification in SI/C and SI/SIII, respectively. **(f)** GSEA is a useful computational method that determines gene ontology and functions. Module genes were imported into GSEA for ontology analysis to exclude module genes with unknown function. At this stage, the number of module genes was 137 and 107 in SI/C and SI/SIII. **(g-h)** Cytoscape is a software platform for visualizing and integrating molecular interaction networks and biological pathways. The filtered module genes were used as seeds to extrapolate networks with 1796 and 1139 genes for exploring 20 and 22 candidate genes with higher level of connectivity in SI/C and SI/SIII respectively in Cytoscape. **(i)** QPCR was used to determine the gene expression of 8 candidate SMA target genes in tissues of type I and III SMA model mice for confirming the regulatory pathways.

**Fig 3:**
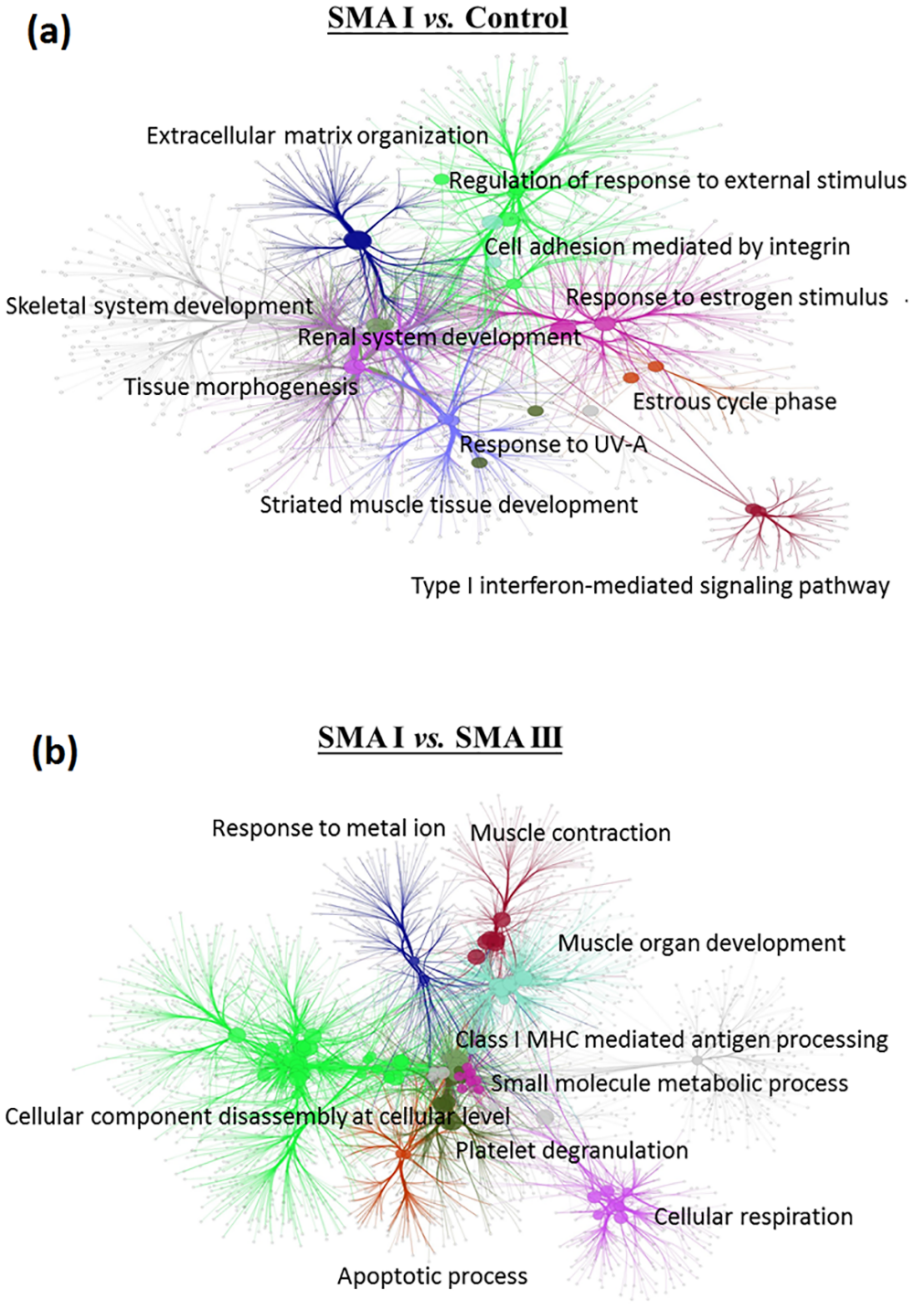
Ontological networks in type I SMA compared to control (SI/C) and in type I SMA compared to type III SMA (SI/SIII). After WGCNA analysis, selected module genes were filtered using GSEA and imported into Cytoscape to reconstruct SMA ontological networks for the two comparison groups. (a) In SI/C, 1796 genes were extrapolated from the 137 module genes by WGCNA analysis. (b) In SI/SIII, 1139 genes were extrapolated from the 107 module genes.

The process of data analysis and gene selection from the data analysis is illustrated in Fig 2. The microarray datasets were categorized into two groups (SI/C and SI/SIII) for exploring the novel targets of SMA. DEGs in SI/C (n = 3304) and SI/SIII (n = 2936) were respectively processed by WGCNA to identify 2 out of 5 modules (r1 = 0.38; r2 = 0.61) in SI/C (n = 213) and 4 out of 17 modules (r1 = 0.64; r2 = 0.58; r3 = 0.37; r4 = 0.24) that were highly correlated with the trait (the difference between SI/C or SI/SIII) in SI/SIII (n = 157). As the module genes with unknown ontology in GSEA were excluded, the remaining module genes of SI/C (n = 137) and SI/SIII (n = 107) were used as seeds to reconstruct gene networks for pathway identification using the Cytoscape plug-in ClueGO+CluePedia. For the SI/C ontological network (Fig 3a), the ClueGO results showed that the reconstructed gene networks (1796 genes) extrapolated from the 137 module genes were involved in several SMA relevant functions such as renal system development, striated muscle tissue development, tissue morphogenesis, skeletal system development, etc. For the SI/SIII ontological network (Fig 3b), 107 module genes were imported into Cytoscape to extend a network of 1139 genes with developmental functions including muscle contraction, muscle organ development, platelet degranulation, etc.

### Target gene identification in SI/C and SI/SIII

**Fig 4:**
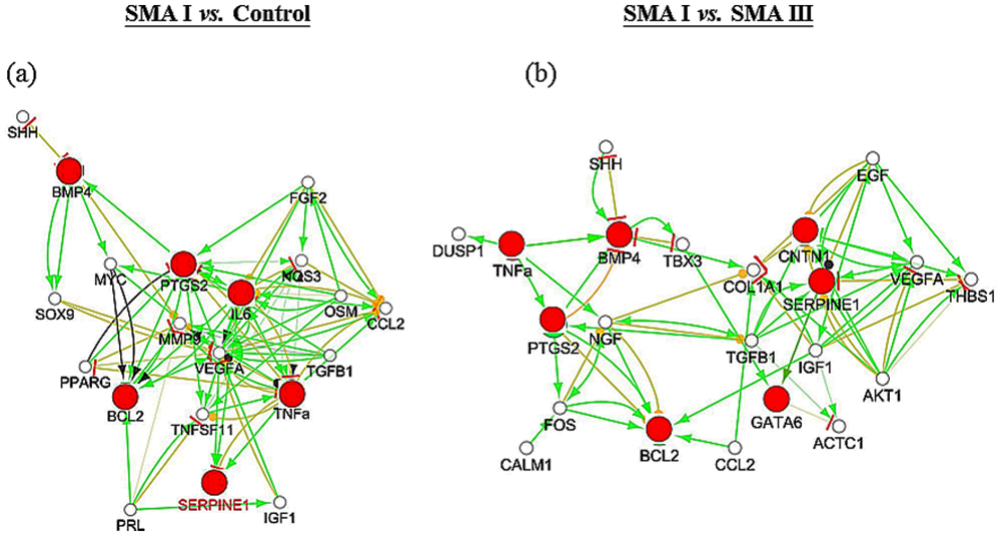
Sub-networks of type I SMA compared to controls (SI/C) and type I SMA compared to type III SMA (SI/SIII). After the reconstruction of SMA ontological networks, several important hub genes were further selected based on the higher connectivity with other genes. (a) In SI/C, 6 out of 20 hub genes were selected as candidate target genes, including *TNFα*, *Bmp4*, *Serpine1*, *Ptgs2*, *Bcl2*, and *IL6*. (b) In SI/SIII, 7 out of 22 hub genes were indicated as candidate target genes, including *TNFα*, *Bmp4*, *Serpine1*, *Gata6*, *Ptgs2*, *Bcl2*, and *Cntn1*. Among these hub genes, several of them have been indicated in SMA in other studies, including *Shh* [29], *Bcl2* [30], *Ccl2* [31], *VEGFA* [32], *Prl* [33], *Igf1* [26], *Fos* [34], *Ngf* [35], and *Egf* [34]. Enlarged red dots, selected candidate target genes; green arrow, activation; yellow line with red bar, inhibition; yellow line with circle, co-expression.

Specific and representative gene connections within each function were merged to create a complex gene network to identify sub-networks for SI/C and SI/SIII. Based on superior connectivity with other genes, we selected 20 SI/C (Fig 4a) and 22 SI/SIII hub genes (Fig 4b) for constructing potential disease-related pathways (Fig 4). Furthermore, candidate SMA targets were filtered from these two sub-networks according to the nodes of initiation, critical connection and termination. For SI/C, 6 out of 20 hub genes were selected as candidate targets including tumor necrosis factor α (*TNFα*), bone morphogenetic protein 4 (*Bmp4*), serpin peptidase inhibitor clade E member 1 (*Serpine1*), prostaglandin-endoperoxide synthase 2 (*Ptgs2*), B-cell CLL/lymphoma 2 (*Bcl2*), and interleukin 6 (*IL6*) (Fig 4a). For SI/SIII, 7 out of 22 hub genes were indicated, including *TNFα*, *Bmp4*, *Serpine1*, GATA binding protein 6 (*Gata6*), *Ptgs2*, *Bcl2*, and contactin 1 (*Cntn1*) (Fig 4b). Therefore, after taking off the duplicates between the two groups, 8 hub genes were selected as candidate SMA targets including *TNFα*, *Bmp4*, *Serpine1*, *Gata6*, *Ptgs2*, *Bcl2*, *IL6 and Cntn1*.

### Gene expression analysis of SMA targets in type I and type III SMA mice

**Fig 5:**
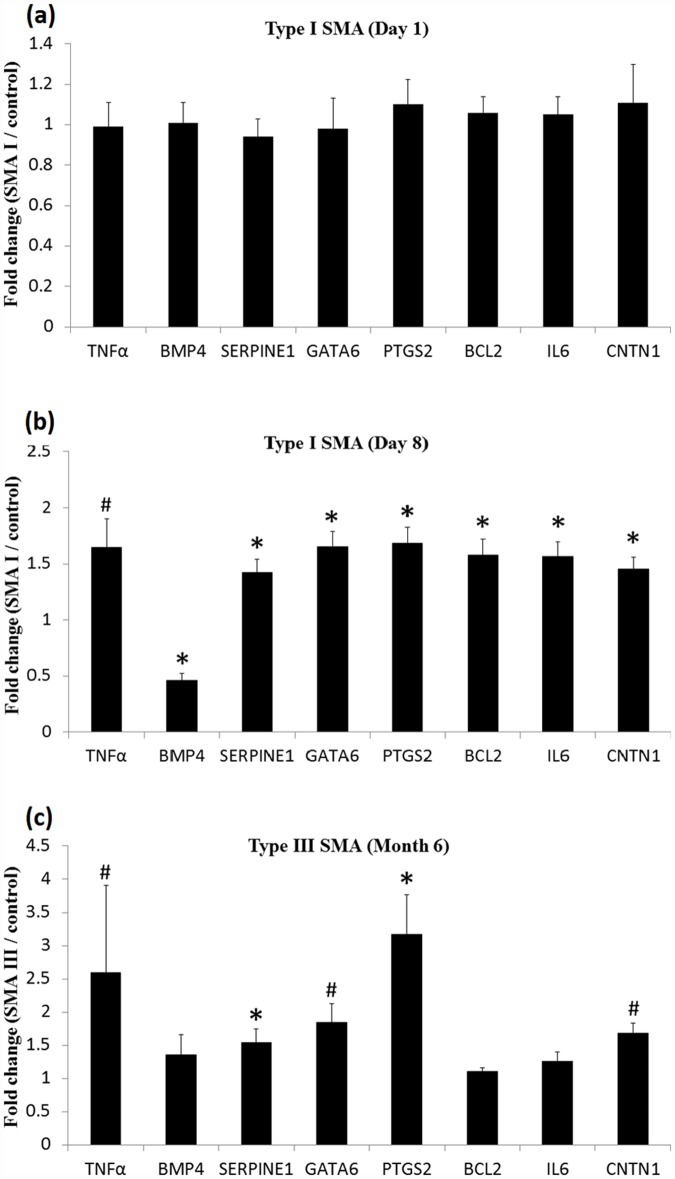
Expression of target genes in the spinal cord of type I and type III SMA mice in different severities. (a) On postnatal day 1 of type I SMA mice (n = 5), all candidate genes presented no different changes; (b) on postnatal day 8 of type I SMA mice (n = 3), all candidate genes expressed significant differences; (c) at the age of 6 months of type III SMA mice (n = 5), *Ptgs2* and *Serpine1* expressed obvious differences. *P < 0.05; ^#^0.05 < P < 0.1. Data are presented as mean of fold-change ± standard deviation.

Analysis of spinal cords of type I (postnatal days 1 and 8) and type III SMA mice (6 months of age) and age-matched heterozygous controls was used to confirm the gene expression of the candidate SMA target genes identified by network analysis. Results showed that none of these genes was significantly altered in type I SMA mice on postnatal day 1 (pre-symptomatic stage) compared with age-matched control littermates (Fig 5a). However, in type I SMA mice on postnatal day 8 (severe symptomatic stage) (Fig 5b), expression of *TNFα* (fold change of mean ± standard deviation 1.65 ± 0.25, P = 0.069) was up-regulated but not statistically significant, that of *Bmp4* (0.46 ± 0.06, P = 0.024) was clearly decreased, and that of *Serpine1* (1.42 ± 0.12, P = 0.021) and *Gata6* (1.65 ± 0.14, P = 0.024) were increased significantly. In addition, the expression of *Ptgs2* (1.68 ± 0.15, P = 0.006), *Bcl2* (1.58 ± 0.14, P = 0.042), *IL6* (1.57 ± 0.13, P = 0.033), and *Cntn1* (1.45 ± 0.11, P = 0.046) were significantly increased. For type III SMA mice at 6 months of age (mild symptomatic stage) (Fig 5c), only the expression level of *Serpine1* (1.54 ± 0.21, P = 0.043) and *Ptgs2* (3.17 ± 0.59, P < 0.001) were significantly increased, while that of *TNFα* (2.59 ± 1.32, P = 0.078), *Gata6* (1.85 ± 0.28, P = 0.054) and *Cntn1* (1.68 ± 0.16, P = 0.092) were elevated but not statistically significant. The expression of *Bmp4* (1.36 ± 0.30, P = 0.174), *Bcl2* (1.10 ± 0.055, P = 0.196), and *IL6* (1.25 ± 0.14, P = 0.315) in type III SMA mice did not differ significantly from that of age-matched controls. These 7 target genes along with *TNFα* were activated in type I SMA mice on postnatal day 8, part of them (*TNFα*, *Serpine1*, *Ptgs2*, *Gata6* and *Cntn1*) were increased in type III SMA mice at the age of 6 months, and all did not obviously differ from controls in type I SMA mice on postnatal day 1 (Table 2).

**Table 2:** Regulation of potential SMA target genes in the spinal cords from different severity and stages of SMA mice.

Stage	TNFα	BMP4	SERPINE1	GATA6	PTGS2	BCL2	IL6	CNTN1
Type I SMA mice on postnatal day 1^a^	Ο	Ο	Ο	Ο	Ο	Ο	Ο	Ο
Type I SMA mice on postnatal day 8^b^	**↑**	↓*	↑*	↑*	↑*	↑*	↑*	↑*
Type III SMA mice at the age of 6 months^c^	**↑**	Ο	↑*	**↑**	↑*	Ο	Ο	**↑**

^a^: Pre-symptomatic stage;

^b^: Severe symptomatic stage;

^c^: Mild symptomatic stage.

Circle: unchanged expression, upward arrow with asterisk: statistically significant up-regulation, upward arrow: up-regulation, but not statistically significant, and downward arrow with asterisk: statistically significant down-regulation.

**Fig 6:**
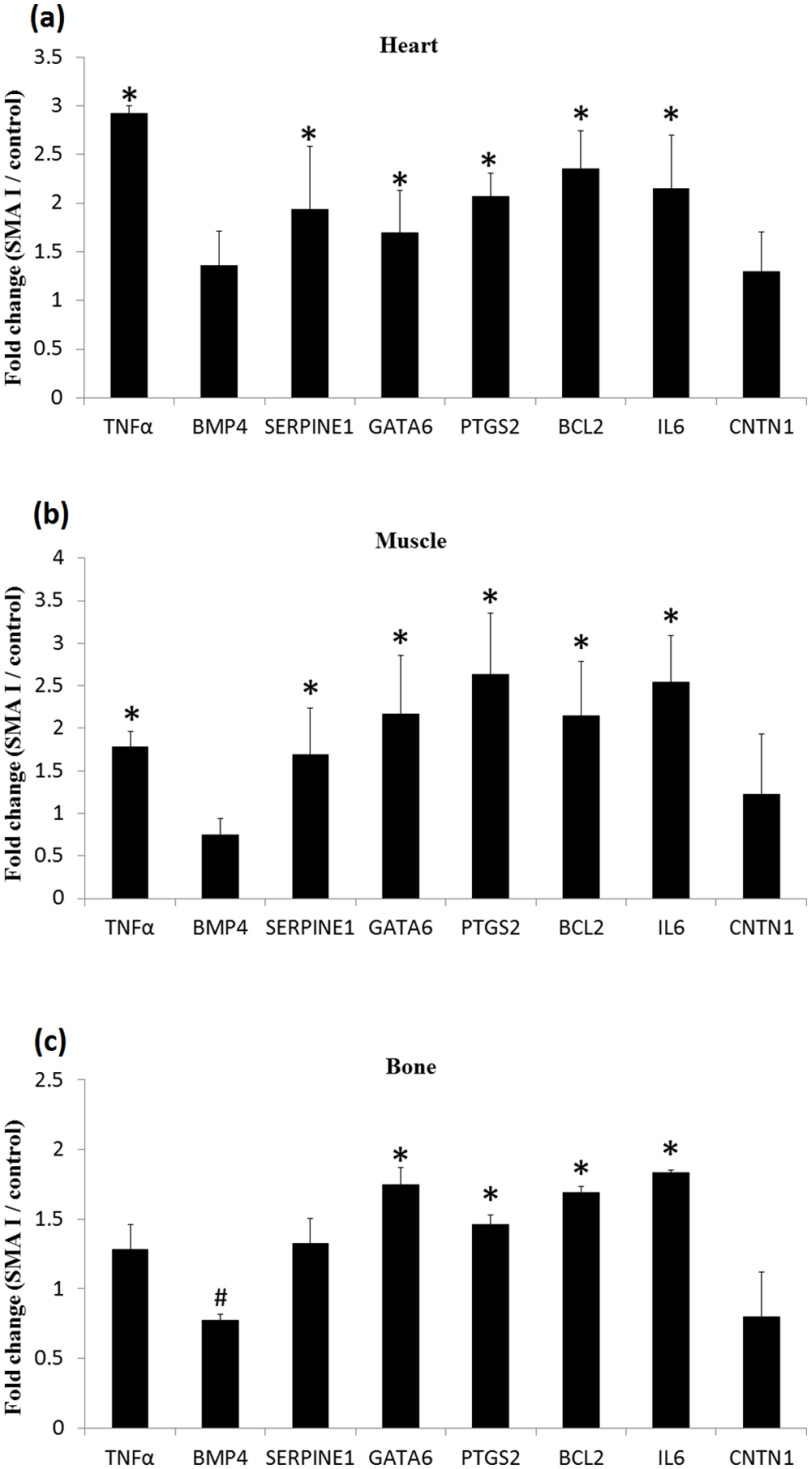
Expression of target genes in heart, muscle and bone tissues in type I SMA mice on postnatal day 8. (a) The expression of *TNFα*, *Serpine1*, *Gata6*, *Ptgs2*, *Bcl2*, and *IL6* in the heart was significantly increased in SMA mice as compared to that in heterozygous control littermates (n = 4). (b) The expression of *TNFα*, *Serpine1*, *Gata6*, *Ptgs2*, *Bcl2*, and *IL6* in the muscles was significantly increased in SMA mice as compared to that in heterozygous control littermates (n = 4). (c) The expression of *Gata6*, *Ptgs2*, *Bcl2*, and *IL6* in the bone tissue was significantly increased in SMA mice as compared to that in heterozygous control littermates (n = 4). *P < 0.05; ^#^0.05 < P < 0.1. Data are presented as the mean fold-change ± standard deviation.

We also determined the gene expression pattern in the heart, muscles and bone from the type I SMA mice on postnatal day 8 since SMA is involved in the malfunction of many organs [16, 17]. Similar to the results collected from the spinal cord, the gene expressions of *Gata6*, *Ptgs2*, *Bcl2* and *IL6* in type I SMA mice were significantly increased in the heart (*Gata6*: 1.70 ± 0.44, P = 0.041; *Ptgs2*: 2.07 ± 0.24, P = 0.011; *Bcl2*: 2.35 ± 0.39, P = 0.005; and *IL6*: 2.15 ± 0.55, P = 0.010), muscle (*Gata6*: 2.17 ± 0.69, P = 0.011; *Ptgs2*: 2.63 ± 0.72, P = 0.022; *Bcl2*: 2.15 ± 0.64, P = 0.011; and *IL6*: 2.54 ± 0.55, P < 0.001), and bone (*Gata6*: 1.75 ± 0.13, P = 0.013; *Ptgs2*: 1.46 ± 0.06, P = 0.042; *Bcl2*: 1.69 ± 0.04, P = 0.042; and *IL6*: 1.84 ± 0.017, P = 0.011) as compared to age-matched heterozygous control littermates (Fig 6). However, the gene expressions of *Serpine1* were significantly upregulated in the heart (1.93 ± 0.65, P = 0.027) and muscles (1.69 ± 0.55, P = 0.046) but not in the bone tissue (1.33 ± 0.18, P = 0.616). The expression of *Bmp4* and *Cntn1* was unaltered in the heart (*Bmp4*: 1.36 ± 0.35, P = 0.214; and *Cntn1*: 1.30 ± 0.41, P = 0.252), muscles (*Bmp4*: 0.75 ± 0.19, P = 0.143; and *Cntn1*: 1.23 ± 0.70, P = 0.863) and bone (*Bmp4*: 0.77 ± 0.04, P = 0.059; and *Cntn1*: 0.80 ± 0.32, P = 0.704) from the SMA mice than that of from the heterozygous control littermates.

### Molecular pathway exploration of SMA symptoms

**Fig 7:**
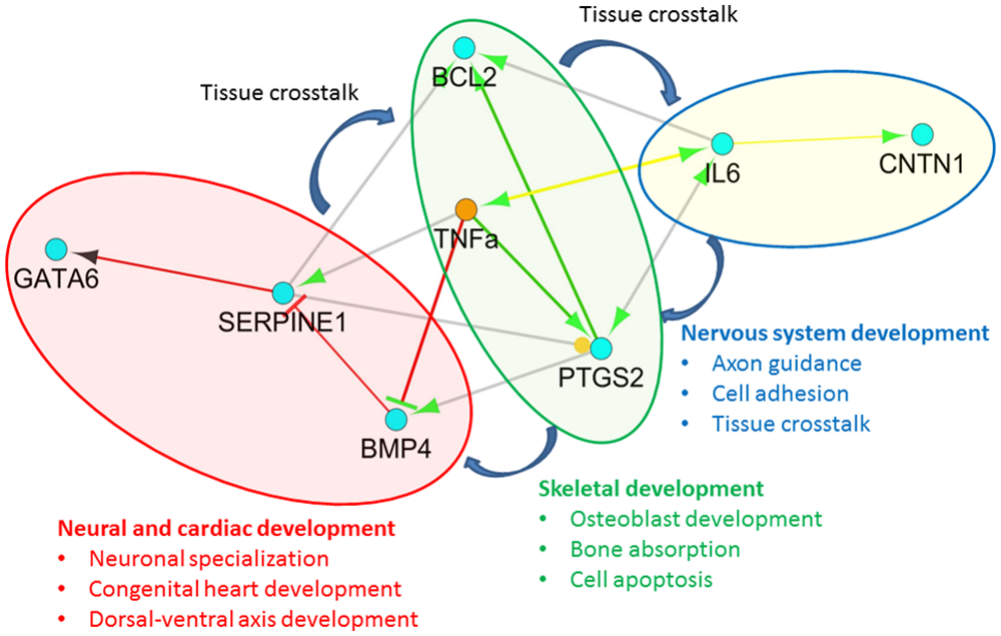
Potential regulatory pathways and functions of candidate genes in SMA. The hub gene *TNFα* is presented as the initial point of 3 pathways: *TNFα*-*Bmp4*-*Serpine1*-*Gata6* in red color (a pathway in neural and cardiac development), *TNFα*-*Ptgs2*-*Bcl2* in green color (a pathway in skeletal development), and *TNFα*-*IL6*-*Cntn1* in yellow color (a pathway in nervous system development). Green arrow, activation; straight line with red bar, inhibition. SI/C, SMA I *vs*. Control; SI/SIII, SMA I *vs*. SMA III.

According to the ontological gene network analysis, these 7 SMA targets along with *TNFα* can be categorized into three biological pathways that was highly correlated with SMA symptom and progression, including the *TNFα-Bmp4-Serpine1-Gata6* pathway (neural and cardiac development), *TNFα-Ptgs2-Bcl2* pathway (skeletal development), and *TNFα-IL6-CNTN1* pathway (nervous system development), based on their major molecular functions (Fig 7).

## Discussion

Our integrative transcriptomic analysis from microarray databases identified 7 target genes as potential SMA targets: *Bmp4*, *Serpine1*, *Gata6*, *Ptgs2*, *Bcl2*, *IL6* and *Cntn1*. These genes are involved in *TNFα* regulation and affect neural [36], cardiac [37], and bone development [38]. Using SMA mouse models to confirm gene expression, we observed that *TNFα* was up-regulated, with attenuated *Bmp4* and increased *Serpine1* and *Gata6* expression (a pathway in neural and cardiac development) during the severe symptomatic stage [39]. *TNFα* up-regulation was also accompanied by elevated levels of *Ptgs2* and *Bcl2* (a pathway in skeletal development) [40], as well as *IL6* and *Cntn1* (a pathway in nervous system development) during the severe symptomatic stage in SMA mice [41]. Since some of the target genes would be affected by development and aging, such as *Bmp4* [42] and *Serpine1* [43], age-matched controls were adopted for eliminating the effect of growth in SMA mouse models.

The pathogenesis of SMA following SMN deficiency is still not fully understood. The existence of modifiers that affect disease progression has been proposed [44]. Previous searches for disease modifiers through microarray analysis provided fruitful information for gene expression comparisons; however, these results were highly variable, likely due to differences in sample tissues and the use of significance testing as a gene list selection method [45, 46]. In the light of our understanding, this is the first SMA study combining gene-gene correlation and molecular network analysis for identifying general SMA targets as potential modifiers. Since SMA involves in multi-system malfunctions, we collected publicly available microarray data from three separate studies comprised various human SMA samples (n = 39) in four distinct human cells (iPS: 12; iPS differentiated motoneurons: 6; fibroblast cells: 3; muscle cells: 18) to perform an integrative transcriptomic analysis across different cell types to identify novel SMA target genes. Although this sample size is the largest undertaken thus far using microarray databases excluding studies of therapeutic agents and is comparatively large among studies of rare diseases [47, 48], 39 samples are relatively small in general meta-analysis to fully support conclusions. Combining gene expression data with GWAS (genome-wide association study) studies might be a suitable way for improving the quality of analysis. To identify potential molecular pathways and candidate genes in SMA pathogenesis, this analysis employed 3 modalities not previously included in SMA studies: WGCNA gene-gene correlation analysis, GSEA gene ontology analysis, and Cytoscape molecular network visualization analysis. From integrated analysis of the 2 comparisons (SMA I *vs*. control; SMA I *vs*. SMA III), we subsequently identified 7 target genes (*Bmp4*, *Serpine1*, *Gata6*, *Ptgs2*, *Bcl2*, *IL6*, and *Cntn1*) potentially relevant to SMA pathogenesis underlying *TNFα* regulation based on the connectivity and biological functions.

These potential SMA target genes were firstly validated in the spinal cords of SMA mice since spinal motor neurons are the primary pathological target in SMA. We firstly confirmed that the expression of GAPDH in each sample is equally expressed in both SMA mice and controls since several housekeeping genes have been found to be differentially expressed in some neurodegenerative diseases in some circumstances at protein level [49, 50]. In type I SMA mice on postnatal day 8 (severe symptomatic stage), 3 biological pathways were activated: *TNFα-Bmp4-Serpine1-Gata6*, *TNFα-Ptgs2-Bcl2*, and *TNFα-IL6-Cntn1*. Notably, gene expression of the target genes within these pathways did not differ from controls in type I SMA mice on postnatal day 1 (pre-symptomatic stage) and was partially altered in type III SMA mice (symptomatic stage of mild SMA). These findings suggest that dysregulation of the implicated pathways correlates with disease stage and severity in SMA mice. We also determined the differential expressed patterns in heart, muscles and bone tissues from type I SMA mice on postnatal day 8 and the results were generally similar with those collected from spinal cords. Further investigation of these molecular pathways is needed to understand the pathological progression of SMA and to provide information for optimizing disease diagnosis and therapy.

In addition to motor neuron degeneration and neuromuscular dysfunction, SMA also features cardiomyopathy and congenital heart defects [51]. In this study of SMA mice, changes in the expression of genes involved in the *TNFα-Bmp4-Serpine1-Gata6* pathway included increased *TNFα*, *Serpine1*, and *Gata6* and decreased Bmp4. BMP4 is a polypeptide belonging to the TGFβ superfamily that contributes to neural and cardiac development during embryogenesis [52]. In xenopus ventralized embryos, down-regulation of *Bmp4* caused severe suppression of early neuronal Wnt signaling in the dorsal ectoderm, interfering with early neuroectoderm specification [53]. BMP-deficient xenopus embryos also have endodermal defects including decreased expression of cardiac-muscle-specific genes and a decrease in the number of cardiomyocytes [54, 55]. SERPINE1 is a serine protease inhibitor that becomes up-regulated in a variety of pathologies including cerebral and myocardial infarction [56]. Increased *Serpine1* expression is associated with accumulation of amyloid peptide in the brains of patients with Alzheimer’s disease [57]. Overexpression of *Gata6*, a zinc finger transcription factor that regulates embryogenesis during vertebrate development, induces hypertrophic cardiomyopathy in mice [58]. Therefore, disruption of the *TNFα-Bmp4-Serpine1-Gata6* pathway might critically suppress neuronal transduction and development and compromise the growth of cardiomyocytes, thereby affecting neuronal and cardiac function in SMA patients.

Another concern for SMA patients is decreased bone density, which manifests clinically as bone remodeling and fractures [59]. Although lack of ambulation might be one of the reasons for the bone loss in some cases, SMA patients have shown an increased incidence of congenital bone fractures and hypercalcaemia caused by altered bone turnover according to clinical observations [60, 61]. The *TNFα-Ptgs2-Bcl2* pathway, which is associated with skeletal development, was activated with increased gene expression levels of *TNFα*, *Ptgs2*, and *Bcl2* in SMA mice in this study. PTGS2 is an inflammatory mediator involved in bone fracture healing, and inhibition of PTGS2 is associated with suppression of bone absorption and osteoporosis [62, 63]. Overexpression of *Bcl2* inhibits osteoblast differentiation to disturb bone remodeling in mice [40]. Therefore, dysregulation of the *TNFα-Ptgs2-Bcl2* pathway might be a cause of the bone loss and osteoporosis observed in SMA patients. BCL2 is also an important anti-apoptotic protein [64]. In *Bcl2* transgenic mice, this protein has been found to be co-expressed with SMN protein to confer a synergistic preventive effect against Bax-induced or Fas-mediated apoptosis. This anti-apoptotic activity of SMN in concert with BCL2 is revoked in the absence of *Smn* underlying the pathogenesis of SMA [65]. Therefore, the *TNFα-Ptgs2-Bcl2* pathway plays a complex role in SMA pathogenesis, affecting neuronal anti-apoptosis and modulating bone formation.

The *TNFα-IL6-Cntn1* pathway is crucial for nervous system development [66, 67]. SMA mice in this study had increased expression levels of the *TNFα-IL6-Cntn1* pathway genes *TNFα*, *IL6*, and *Cntn1*. The interleukin IL6 is also secreted by skeletal muscle as a myokine, serving both paracrine and endocrine roles [68]. Muscles have been reported to engage in crosstalk through the secretion of myokines such as IL6 to communicate from tissue to tissue [69]. CNTN1 is a neuronal membrane protein that forms axon connections in the developing nervous system. Down-regulation of *Cntn1* can cause lethal neurodegenerative phenotypes and congenital myopathy in mice [41]. Conversely, overexpression of *Cntn1*redirects neurites to promote lamina-specific targeting of retinal dendrites in chicks [70]. Based on these observations, *IL6* release might be triggered as a myokine to alert the CNS to increase *Cntn1* expression for neuronal protection in response to the adverse effects of SMN deficiency in SMA mice.

## Conclusions

Microarray cross-cell analysis and verification in a SMA mouse model identified 7 novel target genes (*Bmp4*, *Serpine1*, *Gata6*, *Ptgs2*, *Bcl2*, *IL6* and *Cntn1*) potentially relevant to SMA pathogenesis underlying *TNFα* regulation. These targets belonged to 3 biological pathways: *TNFα-Bmp4-Serpine1-Gata6*, *TNFα-Ptgs2-Bcl2*, and *TNFα-IL6-Cntn1*. These pathways were all activated in mice with severe SMA during the symptomatic stage. Although the roles of these target genes in the pathogenesis of SMA are still unclear, this study provides important information for future research regarding therapeutics and disease mechanisms in SMA.
